# Ten‐year clinical characteristics of patients with early‐onset type 2 diabetes: A single‐center experience in China

**DOI:** 10.1111/1753-0407.13477

**Published:** 2023-09-26

**Authors:** Ruiqi Yu, Xiaoyu Liu, Ruochen Li, Xinhua Xiao

**Affiliations:** ^1^ National Health Commission (NHC) Key Laboratory of Endocrinology, Diabetes Research Center of Chinese Academy of Medical Sciences Department of Endocrinology, Peking Union Medical College Hospital, Peking Union Medical College, Chinese Academy of Medical Sciences Beijing China

**Keywords:** antidiabetic drugs, drug utilization, early‐onset type 2 diabetes, glycemic control

## Abstract

**Aims:**

The incidence of type 2 diabetes in China has exhibited an increasing trend, including younger individuals, over the past years. Early‐onset type 2 diabetes (EOT2D) refers to diabetes diagnosed before 40 years of age. These patients have poor metabolic control and are highly susceptible to diabetic complications, which poses challenges for treatment. However, few studies have reported on the treatment of EOT2D. We determined clinical features and trends in drug use in patients with type 2 diabetes mellitus (T2DM).

**Materials and Methods:**

This retrospective study was performed at the Endocrinology Ward, Peking Union Medical College Hospital (PUMCH). “Diabetes” was used when searching PUMCH's Electronic Medical Record Analytical Database to obtain clinical data of patients between January 2013 and May 2022.

**Results:**

The analysis included 1590 patients with T2DM. Among them, 609 (38.3%) had EOT2D. Compared with late‐onset type 2 diabetes (LOT2D) patients, EOT2D patients exhibited worse glycemic control and higher body weight and lipid levels despite significant age differences. EOT2D patients also had a higher risk of diabetic retinopathy and nephropathy. Under the general trend of increasing use of dipeptidylpeptidase‐4 inhibitors, sodium‐glucose cotransporter‐2 inhibitors, and glucagon‐like peptide‐1 agonists, patients with EOT2D were more likely to use organ‐protective drugs.

**Conclusions:**

Compared with LOT2D patients, EOT2D patients have a longer course of diabetes, worse metabolic control, and a higher rate of developing microvascular complications. The administration of combined therapy, including new agents, may require consideration when selecting hypoglycemic agents for treating EOT2D.

## Graphical abstract

Highlights

- Early‐onset type 2 diabetes refers to diabetes diagnosed before 40 years of age.
- Early‐onset type 2 diabetes patients had worse glycemic control, higher weight and lipid levels, and higher risks of diabetic nephropathy.
- Patients with early‐onset type 2 diabetes were more likely to use organ‐protective drugs.

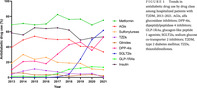

## 1 INTRODUCTION

Over the past decades, diabetes has become a global public health problem. According to the International Diabetes Federation, worldwide, 537 million adults aged 20 to 79 have diabetes, with China accounting for 140.9 million cases.^1^ Type 2 diabetes mellitus (T2DM) is the most common type of diabetes worldwide and accounts for more than 90% of cases.^1^ Although T2DM is an age‐related disease, the prevalence of diabetes among young adults is rising. Early‐onset type 2 diabetes (EOT2D) refers to diabetes diagnosed before 40 years of age.^2^ Recent research has established that from 1990 to 2019, the age‐standardized incidence rate and age‐standardized disability‐adjusted life year rate for the burden of EOT2D increased substantially worldwide.^3^ In clinic‐based settings across Asia, young‐onset diabetes occurred in 20% of adult patients,^2^ and the number of early‐onset diabetes cases has increased fourfold in China from 1997 to 2010.^4^ According to a 2017 nationwide survey, the prevalence of total diabetes in individuals <40 years old in China has reached 3.9%.^5^

Several studies have revealed that there are various risk factors for EOT2D, such as obesity, low physical activity, male sex, and family history of T2DM.^2^, ^3^, ^6^ Patients with EOT2D have a longer duration of hyperglycemia, a more rapid decline in beta cell function, and worse metabolic control.^3^ EOT2D patients are also prone to diabetes‐related complications. The Treatment Options for type 2 Diabetes in Adolescents and Youth (TODAY) study reported that the incidence of diabetic kidney disease, nerve disease, and retinal disease was 54.8%, 32.4%, and 51.0%, respectively.^7^ While the first‐line medication for EOT2D metformin achieves durable glycemic control in only half of the children and adolescents with T2DM, fewer patients received organ‐protective drugs in the EOT2D group than in the late‐onset type 2 diabetes (LOT2D) group.^2^, ^8^ This study set out to compare the clinical features and trends of drug use between patients with EOT2D and LOT2D treated at the Peking Union Medical College Hospital (PUMCH) from January 2013 to May 2022.

## 2 MATERIALS AND METHODS

### 2.1 Study population

Data were obtained from PUMCH's Electronic Medical Record Analytical Database. The inclusion criteria were as follows: the principal diagnosis from the patient's admission and discharge diagnosis was T2DM, and the age at diagnosis was ≥18 years. Patients with T2DM hospitalized at the Endocrinology Ward of PUMCH from January 2013 to May 2022 were enrolled. The diagnosis of diabetes was based on the World Health Organization's criteria for diagnosis and classification.^9^ Exclusion criteria included secondary diabetes mellitus, such as following pancreatectomy, Cushing's syndrome, and pheochromocytoma. Ultimately, 1590 eligible patients with T2DM were included in the analysis.

### 2.2 Data collection

The following information was gathered through a retrospective analysis of the patients' clinical data: age, sex, age at diabetes onset, diabetes duration, family history of diabetes, laboratory test results, and pharmacological management regimens. For patients with multiple admissions during the study period, we selected the last admission. The diagnosis of diabetic nephropathy, diabetic retinopathy, diabetic neuropathy, hypertension, or hyperlipidemia was based on the patients' discharge diagnoses. Pharmacological management regimens were defined as prescriptions from discharge summaries and drug orders.

### 2.3 Definitions

The age at onset was calculated based on the difference between the duration of the disease and the patient's age. Hospitalized adult patients with T2DM were classified as EOT2D if they were diagnosed before age 40 and as LOT2D if they were diagnosed after or at 40 years of age. To evaluate control of glycemic and low‐density lipoprotein cholesterol (LDL‐C), we set the cutoff for glycated hemoglobin (HbA1c) and LDL‐C at 7.0% and 2.6 mmol/L respectively. Diabetic retinopathy was assessed by direct funduscopic examination. Diabetic neuropathy was determined according to the existence of typical symptoms. Microalbuminuria was defined as an albumin‐to‐creatinine ratio (ACR) between 30 and 300 mg/g or a urinary albumin excretion rate (UAER) between 20 and 200 μg/min; macroalbuminuria was defined as an ACR >300 mg/g or a UAER >200 μg/min. The nine classes of glucose‐lowering drugs used during the study period were metformin, sulfonylureas, α‐glucosidase inhibitors (AGis), thiazolidinediones (TZDs), dipeptidyl peptidase‐4 inhibitors (DPP‐4is), glinides, glucagon‐like peptide‐1 receptor agonists (GLP‐1RAs), sodium‐glucose cotransporter‐2 inhibitors (SGLT2is), and insulin. Insulin administration was defined as the use of any long‐, medium‐ or short‐acting insulin.

### 2.4 Laboratory assessment

HbA1c was measured by a D100 HPLC instrument‐reagent system (Bio‐Rad, Hercules, California). A Roche C8000 automatic analyzer (Roche, Basel, Switzerland) was used to measure serum total cholesterol (TC), triglyceride (TG), high‐density lipoprotein cholesterol (HDL‐C), LDL‐C, and glucose. All laboratory analyses were completed at the laboratory department of PUMCH.

### 2.5 Statistical analysis

Continuous data are shown as the mean ± SD. Categorical variables are expressed as numbers and percentages. Comparisons were made by Student's *t* test or the chi‐square test. Binary logistic regression models were performed using the enter method. A univariate logistic regression model analysis was fitted prior to multivariate regression models. The proportion of missing data associated with each of the following variables is shown in parentheses: HbA1c (0.5%), TC (0.1%), TGs (0.1%), LDL‐C (0.1%), HDL‐C (0.3%), and ACR (1.3%). Mean imputation methods were performed to treat these missing data. Statistical analyses were carried out using IBM SPSS Statistics for Windows version 26 (IBM Corp., Armonk, NY, USA). A two‐tailed *p* < .05 indicated statistical significance.

## 3 RESULTS

### 3.1 Characteristics of the patients

The mean age of the 1590 patients with T2DM was 55.6 years, and 58.8% were male. A total of 609 (38.3%) patients had EOT2D, with a mean age at diagnosis of 32.2 years (SD: 5.3) compared to 49.6 years (SD: 7.6) for patients with LOT2D (*n* = 981). Patients with EOT2D were more likely to be younger and to have a longer diabetic duration (13.4 years vs. 12.1 years), a family history of diabetes (75.0% vs. 63.1%), and a higher body mass index (BMI) than those with LOT2D. Significant differences regarding drinking and smoking history were also found, with both factors being more frequent in EOT2D than in LOT2D (Table 1).

**TABLE 1:** Clinical characteristics of patients with EOT2D vs LOT2D.

Variables	EOT2D (*n* = 609)	LOT2D (*n* = 981)	*p* value
	Mean/number (SD or %)	Mean/number (SD or %)	
Age, years	45.6 (11.6)	61.7 (8.8)	<.001
Age at diabetes diagnosis, years	32.2 (5.3)	49.6 (7.6)	<.001
Male sex	390 (64.0%)	545 (55.6%)	.001
Duration of diabetes, years	13.4 (9.2)	12.1 (7.5)	<.001
Hospitalization days	11.4 (6.4)	11.6 (5.7)	.354
Family history			<.001
Positive	457 (75.0%)	619 (63.1%)	
Negative	152 (25.0%)	362 (36.9%)	
Smoking history			.001
Positive	280 (46.0%)	370 (37.7%)	
Negative	329 (54.0%)	611 (62.3%)	
Drinking history			.013
Positive	359 (58.9%)	516 (52.6%)	
Negative	250 (41.1%)	465 (47.4%)	

*Note*: *p* values were derived using the chi‐square test or Student *t* test.

Abbreviations: EOT2D, early‐onset type 2 diabetes; LOT2D, late‐onset type 2 diabetes.

### 3.2 Multivariate analysis of EOT2D risk

The following factors were included in the final calculation: sex, smoking and drinking history, and family history of diabetes. The results showed that family history (odds ratio [OR] 1.80, 95% confidence interval [CI] 1.44–2.26) was a risk factor for EOT2D (*p* < .001). The results of the logistic regression analysis are presented in Figure S1 and Table S1.

### 3.3 Patients' glycemic control, lipid profile, and BMI levels

The results showed that EOT2D patients had significantly poorer glycemic control, with a higher average HbA1c and a lower proportion of patients with HbA1c <7%. The EOT2D group also had significantly lower HDL‐C, higher TG, and higher TC concentrations (Table 2). LDL‐C concentrations and proportion of patients with LDL‐C <2.6 mmol/L did not differ significantly among groups before adjusting for disease duration and sex. EOT2D patients also had higher rates of obesity and fewer patients with BMI < 24 kg/m^2^. BMI decreased with age at diagnosis of T2DM. The average BMI was 28.03 kg/m^2^ in the youngest age group, whereas in the oldest age group, it was 25.09 kg/m^2^ (Figure S2).

**TABLE 2:** Glycemic control, lipid profile, and BMI level in patients with EOT2D and LOT2D.

Clinical index	EOT2D (*n* = 609) Mean/number (SD or %)	LOT2D (*n* = 981) Mean/number (SD or %)	Unadjusted *p* value	Adjusted *p* value *
HbA1_c_, %	8.9 (2.0)	8.7 (2.0)	.006	.002
TC, mmol/L	4.8 (1.4)	4.5 (1.2)	<.001	<.001
LDL‐C, mmol/L	2.7 (1.0)	2.6 (0.9)	.088	.008
HDL‐C, mmol/L	1.1 (0.3)	1.1 (0.3)	.009	.027
HbA1c <7%	89 (14.6%)	204 (20.8%)	.002	.002
LDL‐C <2.6 mmol/L	292 (47.9%)	498 (50.8%)	.275	.049
BMI, kg/m^2^	26.8 (4.7)	25.7 (3.7)	<.001	<.001
Overweight	242 (39.7%)	430 (43.8%)	.108	.118
Obesity	209 (34.3%)	233 (23.8%)	<.001	<.001
BMI <24 kg/m^2^	158 (25.9%)	318 (32.4%)	.006	.006

*Note*: BMI = body mass [kg]/(body height [m^2^]).^2^ Overweight was defined as BMI ≥ 24.0 kg/m^2^ but <28.0 kg/m^2^, and obesity was defined as BMI ≥ 28.0 kg/m^2^. *p* values were derived by using the chi‐square test or Student *t* test.

Abbreviations: BMI, body mass index; EOT2D, early‐onset type 2 diabetes; HbA1c, glycated hemoglobin; HDL‐C, high‐density lipoprotein cholesterol; LDL‐C, low‐density lipoprotein cholesterol; LOT2D, late‐onset type 2 diabetes; TC, total cholesterol; TG, triglycerides.

* Adjusted for disease duration and sex.

### 3.4 Occurrence of diabetes comorbidities and complications

Although there were no significant differences between the groups regarding serum creatinine level and incidence of albuminuria, patients with EOT2D had higher rates of diabetic nephropathy and diabetic retinopathy than patients with LOT2D. This association between EOT2D and diabetic nephropathy remained significant after adjusting for age, sex, and disease duration. The proportions of hypertension were significantly higher in patients with LOT2D (Table 3).

**TABLE 3:** Occurrence of diabetes comorbidities and complications in patients with EOT2D and LOT2D.

Variables	EOT2D (*n* = 609)	LOT2D (*n* = 981)	*p* value	*p* value*
	Mean/number (SD or %)	Mean/number (SD or %)		
Complication				
Diabetic nephropathy	187 (30.7%)	217 (22.1%)	<.001	.014
Cr (E)	75.3 (43.8)	73.3 (28.6)	.279	.106
Microalbuminuria	116 (19.0%)	165 (16.8%)	.258	.240
Macroalbuminuria	86 (14.1%)	99 (10.1%)	.015	.733
Diabetic retinopathy	238 (39.1%)	280 (28.5%)	<.001	.348
Diabetic neuropathy	198 (32.5%)	332 (33.8%)	.584	.338
Comorbidity				
Hypertension	298 (48.9%)	622 (63.4%)	<.001	<.001
Hyperlipidemia	429 (70.4%)	728 (74.2%)	.101	.099

*Note*: *p* values were derived by using the chi‐square test or Student *t* test. *p* value*, adjusted for sex, age, and duration of diabetes.

Abbreviations: Cr, creatinine; EOT2D, early‐onset type 2 diabetes; LOT2D, late‐onset type 2 diabetes.

### 3.5 Changes in prescription patterns of antidiabetic medications

**FIGURE 1:**
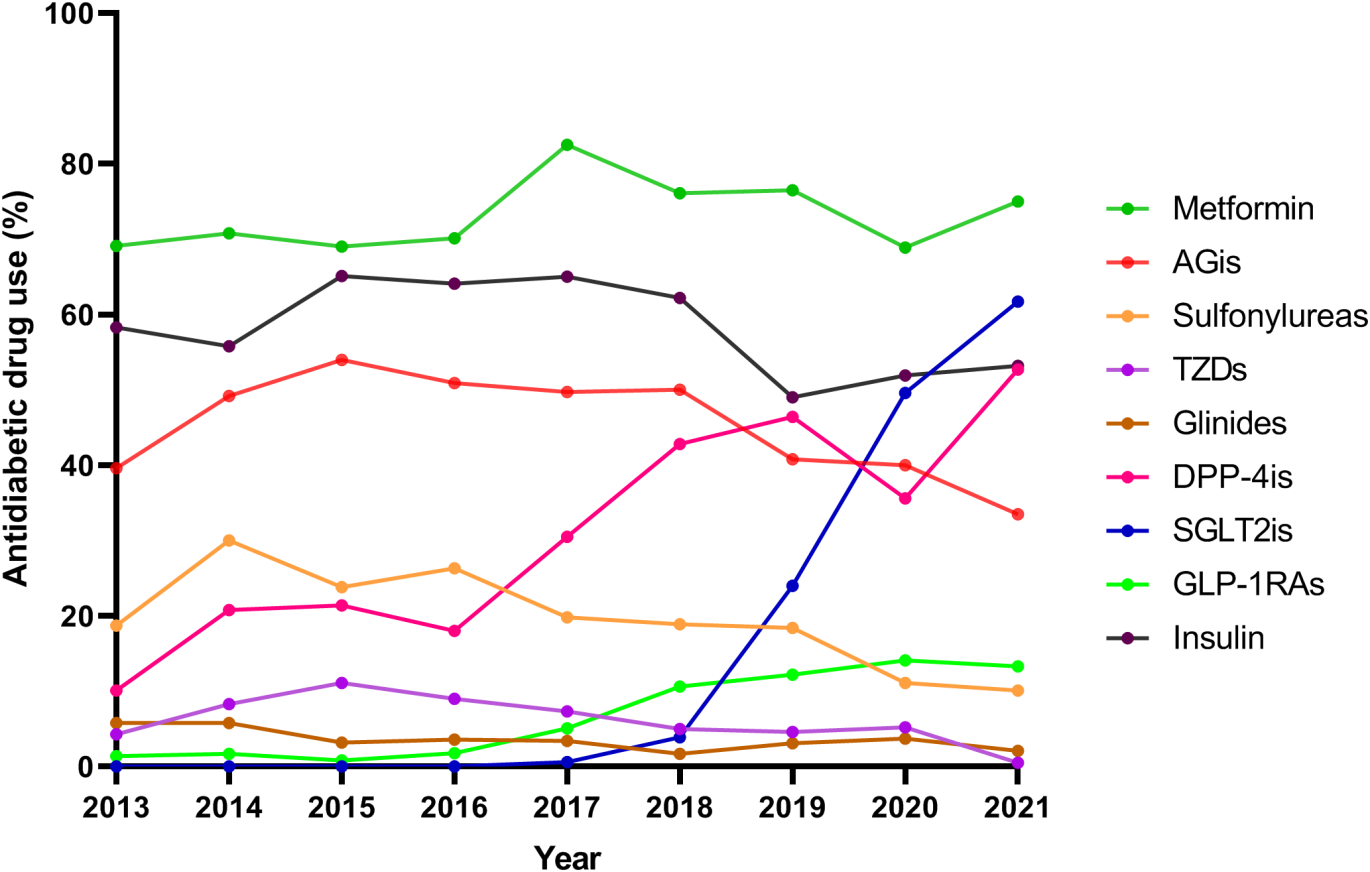
Trends in antidiabetic drug use by drug class among hospitalized patients with T2DM, 2013–2021. AGis, alfa glucosidase inhibitors; DPP‐4is, dipeptidylpeptidase 4 inhibitors; GLP‐1RAs, glucagon‐like peptide 1 agonists; SGLT2is, sodium glucose co‐transporter 2 inhibitors; T2DM, type 2 diabetes mellitus; TZDs, thiazolidinediones.

From 2013 to 2021, there was an increase in the use of SGLT2is, GLP‐1RAs, and DPP‐4is and a decrease in use of traditional agents such as sulfonylureas. Metformin was the most commonly prescribed oral medication from 2013 to 2021. AGis, with a rate of 39.6%, were the second most commonly used oral medication among patients with T2DM who received glucose‐lowering therapy in 2013, which decreased to 33.5% in 2021. The use of glucose‐lowering medications with established cardiovascular benefits, including GLP‐1RAs and SGLT2is, started to rise from 2016 and 2018 to 13.3% and 61.7% in 2021, respectively. DPP‐4i users increased dramatically from 2016 and composed of 52.7% of the users of hypoglycemic agents in 2021. The proportion of TZD and glinide users remained low over time (Figure 1, Table S2).

**FIGURE 2:**
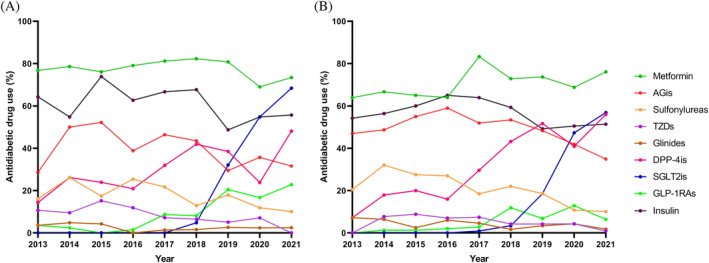
Trends in antidiabetic drug use by drug class among hospitalized patients in the EOT2D (A) and LOT2D (B) groups, 2013–2021. AGis, alfa glucosidase inhibitors; DPP‐4is, dipeptidylpeptidase 4 inhibitors; EOT2D, early‐onset type 2 diabetes; GLP‐1RAs, glucagon‐like peptide 1 agonists; LOT2D, late‐onset type 2 diabetes; SGLT2is, sodium glucose co‐transporter 2 inhibitors; TZDs, thiazolidinediones.

Patients with EOT2D were more likely to use metformin, TZDs, SGLT2is, GLP‐1RAs, and insulin, whereas those with LOT2D were more likely to use AGis, sulfonylureas, and glinides. There were significantly more patients with EOT2D using metformin and TZDs between 2013 and 2017 and more patients using AGis, DPP‐4is, SGLT2is, and GLP‐1RAs from 2018 to 2022 (*p* < .05) (Table S3). The percentage of patients with EOT2D using DPP‐4is was outnumbered by that of patients with LOT2D in 2018. The increase in SGLT2i users was initially slow but became evident after 2018, and the changes were more pronounced in patients with EOT2D (Table S4, Figure 2).

### 3.6 Combination therapy in T2DM

The proportion of patients using three or more drugs has steadily increased from 23.7% in 2013 to 69.7% in 2021. Meanwhile, the proportions of cases with monotherapy and dual therapy decreased from 24.5% and 51.1% in 2013 to 8.0% and 21.8% in 2021, respectively (Figure S3). No significant difference in monotherapy or combined therapy was found between groups (Table S5). In 2013, insulin combined with metformin was the most frequently used combined therapy (25.0%), followed by insulin with AGis, which accounted for 13.5% of all combination prescriptions. However, metformin with DPP4‐is and SGLT2is was the most commonly prescribed combination therapy in 2021 (9.3%) (Table S6).

## 4 DISCUSSION

According to this retrospective analysis, patients with EOT2D were more likely to have a longer duration of diabetes and poorer glycemic control and to be at greater risk for developing diabetic complications.

Early‐onset diabetes accounted for 23.3% of newly diagnosed diabetes in China, according to a national cross‐sectional survey of 2801 participants.^10^ According to the China Noncommunicable Disease Surveillance 2010 survey, participants with newly diagnosed diabetes between the ages of 18 and 40 composed of 15.7% of all newly diagnosed diabetes patients as determined by the oral glucose tolerance test.^4^ In this study, 609 hospitalized patients (38.3%) with T2DM were diagnosed before 40 years of age. The fact that most of these patients were from Beijing which has the highest obesity incidence in China may explain the high prevalence of EOT2D.^11^

EOT2D results from a complex combination of genetic and environmental factors, including male sex, obesity, family history of T2DM, and low physical activity.^6^ Previous research indicates that those with a family history of diabetes have impaired insulin sensitivity at an early age,^12^ and first‐degree relatives of patients with EOT2D are more likely to develop diabetes.^13^ In this study, we found that patients in the EOT2D group had a higher proportion of males (64% vs 55.6%) and a higher family history of diabetes (75.0% vs 63.1%) than those in the LOT2D group, which was consistent with the findings of these previous studies.^2^ The ratio of patients with tobacco or alcohol use was also higher in the EOT2D group in this study.

Obesity is the leading risk factor for EOT2D. The Joint Asia Diabetes Evaluation (JADE) study enrolled 41 029 patients with type 2 diabetes between 2007 and 2012 and showed that EOT2D patients were more likely to be obese, with an average BMI of 26.5 kg/m^2^ vs 25.7 kg/m^2^, compared with LOT2D patients.^2^ Contrary to these findings, the China National Diabetes and Metabolic Disorders Study has shown that newly diagnosed diabetes participants in China have similar BMIs across different age groups.^10^ In a cross‐sectional survey of the China National HbA1c Surveillance System (CNHSS), the proportions of obesity were significantly higher in the early‐onset group (12% vs 10%, *p* < .0001).^14^ Our results demonstrate that patients with EOT2D have a significantly higher BMI (26.8 vs 25.7, *p* < .01) and higher rates of obesity, with a lower proportion of patients having a BMI lower than 24 kg/m^2^, which supports the findings from previous studies. We also noted a steady decrease in BMI in parallel with increasing age at diabetes diagnosis. Weight management is an important part of T2DM management, and weight loss is associated with improved glucose and lipid metabolism and even sustained remission of T2DM.^15^ Therefore, higher BMI and obesity might be the reason for the poor glycemic control and high prevalence of lipid profile abnormalities in patients with EOT2D.

The JADE study showed that patients with early‐onset diabetes have higher levels of HbA1c and LDL‐C, suggesting that young age at diagnosis is independently associated with poor metabolic profile control.^2^ However, the CNHSS cross‐sectional survey showed that EOT2D patients had lower LDL‐C, higher HbA1c, and similar TG levels.^14^ In our study, only 14.6% of patients with EOT2D had an HbA1c less than 7.0%, and 47.9% had an LDL‐C less than 2.6 mmol/L. Patients with EOT2D were more likely to lead busy lifestyles and work long hours in the first few years after diagnosis, which may have resulted in their inability to use medications on time or visit the clinic regularly. They might also have had poorer awareness of the need to seek medical care. Taken together, all of these factors may have resulted in poor glycemic control and the progression of diabetes complications.

Previous studies have shown that EOT2D is associated with an increased risk of complications because of the long disease duration and exposure to a hyperglycemic environment.^16^ A survey of 29 442 patients with T2DM in China conducted in 2011 showed that EOT2D significantly increased the risk of microvascular diseases, including diabetic nephropathy and diabetic retinopathy, at all ages, especially from 45 years of age and above. These associations were attenuated or disappeared after correction for the duration of diabetes, suggesting that the risk increased with the duration of diabetes.^17^ In our study, the risks of diabetic nephropathy and diabetic retinopathy were both higher in patients with EOT2D, but adjusting for age, sex, and duration of diabetes greatly attenuated the risk of diabetic retinopathy. Contrary to earlier findings,^18^ there was no significant difference in the prevalence of diabetic neuropathy in this study. A possible explanation is that an accurate diagnosis of diabetic neuropathy is difficult because this diagnosis primarily depends on the clinician's judgment according to the patient's symptoms.^19^

Patients with EOT2D have worse metabolic control and a more rapid decline in islet function and are more likely to develop diabetic complications, posing a challenge in treatment. Currently, there are no treatment methods that show obvious advantages in EOT2D. Metformin is recommended as a first‐line medication for T2DM in China; however, in the TODAY study, monotherapy with metformin achieved durable glycemic control in only half of children and adolescents with T2DM.^8^ Meanwhile, the Restoring Insulin Secretion (RISE) pediatric study found that neither long‐acting insulin followed by metformin nor metformin alone preserved β‐cell function in youths.^20^ With the emergence of new antidiabetic drugs in recent years, there have been more treatment options for EOT2D. We summarize the drugs used in hospitalized T2DM patients in PUMCH in recent years and aim to provide evidence to formulate effective therapeutic regimens for EOT2D.

We found considerable and significant changes in the prescription patterns for T2DM in PUMCH inpatients during the previous years with the emergence of new hypoglycemic agents. Metformin has been the most frequently used oral glucose‐lowering drug for the treatment of T2DM in both groups over the last few years. The use of sulfonylureas and AGis, which were used significantly more for LOT2D in 2013, steadily declined over the years. There were no significant differences in insulin use between groups, and we noted stable usage for years. There has been a rapid increase in the number of people with LOT2D using DPP‐4is since 2016, exceeding the number of people with EOT2D who are using DPP‐4is. The use of SGLT2is slowed following a sharp increase in 2018–2020, which was more pronounced in people with EOT2D. Although the use of GLP‐1RAs gradually rose over time, the percentage of LOT2D patients treated with this organ‐protective drug was relatively low (6.4% vs. 22.8%) in 2021.

SGLT2is became available in China in 2017, and GLP‐1RAs were introduced in China in 2009. However, health insurance coverage for GLP‐1RAs was available only for patients with high BMI. Multiple studies have validated the cardiovascular risk reduction with the use of SGLT2is and GLP‐1RAs, which are recommended by diabetes and cardiovascular disease guidelines.^21^, ^22^ CAPTURE, a study of 9832 patients with T2DM across 13 countries in 2019, found that the presence or absence of a history of cardiovascular disease did not affect the proportion of patients using SGLT2is and GLP‐1RAs.^23^ The DISCOVER (Discovering Treatment Reality of Type 2 Diabetes in Real World Settings) study revealed that younger age, male sex, and higher BMI were associated with a greater use of SGLT2is or GLP‐1RAs (16.1%).^24^ In this study, we found that the use of SGLT2is and GLP‐1RAs has increased over time from 2013 to 2021, with particularly high usage among patients with EOT2D, characterized by younger age, higher BMI, and a higher proportion of males. A possible explanation for this might be that risks of genitourinary tract infections, volume depletion, and a high BMI criterion may limit its application in the LOT2D group.

The guidelines for the prevention and treatment of T2DM in China recommended lifestyle intervention and metformin as the first‐line treatments for patients with T2DM. In patients unable to attain glycemic targets with monotherapy, combination therapy with two or three different types of oral hypoglycemic agents should be used.^19^ The VERIFY (Vildagliptin Efficacy in Combination with Metformin for Early Treatment of Type 2 Diabetes) trial shows that compared with monotherapy, early combination therapy of metformin with vildagliptin, a once‐daily DPP4 inhibitor, can reduce the probability of initial treatment failure and result in more patients achieving the target blood glucose level.^25^ In our study, we noted that the number of patients using combination therapy was increasing yearly. Among patients with T2DM in 2013, those receiving monotherapy accounted for 24.5% of all patients. In 2021, only 8.0% were treated with monotherapy, and 69.7% were treated with three or more kinds of drugs simultaneously. In patients using combined therapy in 2021, most cases (80.8%) included the use of metformin, followed by SGLT2is (66.2%) and DPP‐4is (57.5%).

This study has several limitations. One major limitation of our study is selective bias. As PUMCH is a major referral center for complicated patients, PUMCH patient conditions tend to be more complicated than those at other hospitals. Second, the patients in this study were all inpatients. Patients with milder conditions may not be included, and patients' willingness to be admitted to the hospital may also bias the study. Third, because of the difficulty of differential diagnosis, maturity‐onset diabetes of the young or maternally inherited diabetes and deafness may be misdiagnosed as T2DM in this study. Therefore, our sample was not representative of the Chinese diabetes population. Notwithstanding the relatively limited sample, this work offers valuable insights into the clinical features and medication type characteristics of EOT2D in Chinese populations.

## 5 CONCLUSION

The increasing population with EOT2D in China calls for attention. In our retrospective study performed at PUMCH, patients with EOT2D exhibited worse metabolic control and were prone to developing microvascular complications. The administration of combined therapy, including new hypoglycemic agents, may need to be considered when selecting hypoglycemic agents for treating EOT2D. This study emphasizes the importance of diabetes screening among younger individuals with high risks and the annual screening of diabetes complications in EOT2D. Longer follow‐up is needed to explore the effects of different therapies in future studies.

## AUTHOR CONTRIBUTIONS

Ruiqi Yu was responsible for the study design and data analysis and drafted the manuscript. Ruochen Li and Xiaoyu Liu helped with the data collection. Xinhua Xiao contributed to the whole study design and reviewed the manuscript. All authors approved the final version of this manuscript.

## FUNDING INFORMATION

This work was supported by grants from the National High Level Hospital Clinical Research Funding (2022‐PUMCH‐C‐019, 2022‐PUMCH‐B‐121), National Natural Science Foundation of China (No. 82170854, 81870579, 81870545, 81570715, 81170736), Beijing Natural Science Foundation (7202163), Beijing Municipal Science & Technology Commission (Z201100005520011), and CAMS Innovation Fund for Medical Sciences (CIFMS2021‐1‐I2M‐002).

## CONFLICT OF INTEREST STATEMENT

The authors declare that they have no conflict of interest.

## Supporting information


**Figure S1.** Multivariate logistic regression analysis of risk factors of early‐onset type 2 diabetes (EOT2D).
**Figure S2.** Relation of body mass index (BMI) and age at diagnosis among hospitalized patients with type 2 diabetes mellitus (T2DM).
**Figure S3.** Changes in the proportion of patients on monotherapy and combined therapy.

**Table S1.** Risk factors of early‐onset type 2 diabetes (EOT2D).
**Table S2.** Annual prevalence of glucose‐lowering medications used in hospitalized patients with type 2 diabetes mellitus (T2DM).
**Table S3.** Glucose‐lowering medications used in patients with early‐onset type 2 diabetes (EOT2D) and late‐onset type 2 diabetes (LOT2D).
**Table S4.** Comparison of medication use between early‐onset type 2 diabetes (EOT2D) and late‐onset type 2 diabetes (LOT2D), 2013–2021.
**Table S5.** Monotherapy or combined therapy in early‐onset type 2 diabetes (EOT2D) and late‐onset type 2 diabetes (LOT2D) groups.
**Table S6.** Comparison of combined therapy used in 2013 and 2021.

## Data Availability

The data sets used in the current study are not publicly available, but are available from the corresponding author on reasonable request.
