# Supplementary material for: Ten‐year clinical characteristics of patients with early‐onset type 2 diabetes: A single‐center experience in China
Source: J Diabetes. 2023 Sep 26;16(1):e13477. doi: 10.1111/1753-0407.13477 (PMC10809291; doi:10.1111/1753-0407.13477)
Supplement: Supplementary file 1 — Figure S1. Multivariate logistic regression analysis of risk factors of early‐onset type 2 diabetes (EOT2D). Figure S2. Relation of body mass index (BMI) and age at diagnosis among hospitalized patients with type 2 diabetes mellitus (T2DM). Figure S3. Changes in the proportion of patients on monotherapy and combined therapy. [file JDB-16-e13477-s002.docx]

Supplementary Materials

Figure legends

Supplementary Figure 1. Multivariate logistic regression analysis of risk factors of EOT2D.

OR, odds ratio; CI, confidence interval.

Supplementary Figure 2 Relation of BMI and age at diagnosis among hospitalized patients with T2DM.

BMI, Body mass index

Supplementary Figure 3 Changes in the proportion of patients on monotherapy and combined therapy

**Supplementary Figure 1. Multivariate logistic regression analysis of risk factors of EOT2D.**

EOT2D, early-onset type 2 diabetes; OR, odds ratio; CI, confidence interval.

**Supplementary Figure 2 Relation of average BMI and age at diagnosis among hospitalized patients with T2DM.**

BMI, Body mass index

**Supplementary Figure 3 Changes in the proportion of patients on monotherapy and combined therapy**
