# Supplementary material for: Ten‐year clinical characteristics of patients with early‐onset type 2 diabetes: A single‐center experience in China
Source: J Diabetes. 2023 Sep 26;16(1):e13477. doi: 10.1111/1753-0407.13477 (PMC10809291; doi:10.1111/1753-0407.13477)
Supplement: Supplementary file 2 — Table S1. Risk factors of early‐onset type 2 diabetes (EOT2D). Table S2. Annual prevalence of glucose‐lowering medications used in hospitalized patients with type 2 diabetes mellitus (T2DM). Table S3. Glucose‐lowering medications used in patients with early‐onset type 2 diabetes (EOT2D) and late‐onset type 2 diabetes (LOT2D). Table S4. Comparison of medication use between early‐onset type 2 diabetes (EOT2D) and late‐onset type 2 diabetes (LOT2D), 2013–2021. Table S5. Monotherapy or combined therapy in early‐onset type 2 diabetes (EOT2D) and late‐onset type 2 diabetes (LOT2D) groups. Table S6. Comparison of combined therapy used in 2013 and 2021. [file JDB-16-e13477-s001.docx]

Supplementary Materials

Table legends

Supplementary Table 1 Risk factors of EOT2D

Supplementary Table 2 Annual prevalence of glucose-lowering drug used hospitalized patients with T2DM

Supplementary Table 3 Glucose-lowering medications used in patients with EOT2D and LOT2D

Supplementary Table 4 Comparison of medication use between EOT2D and LOT2D, 2013-2021

Supplementary Table 5 Monotherapy or combined therapy in EOT2D and LOT2D groups

Supplementary Table 6. Comparison of combined therapy used in 2013 and 2021

**Supplementary Table 1 Risk factors of EOT2D**

|  | P value | OR (95% CI) |
| --- | --- | --- |
| Gender | 0.079 | 1.28 (0.97-1.69) |
| Smoking | 0.300 | 1.16 (0.88-1.52) |
| Family history | <0.001 | 1.80 (1.44-2.26) |
| Drinking | 0.446 | 1.10 (0.86-1.42) |

Abbreviations: EOT2D, early-onset type 2 diabetes. OR, odds ratio; CI, confidence interval.

**Supplementary Table 2 Annual prevalence of glucose-lowering drug used hospitalized patients with T2DM, 2013-2021**

| Year | TCN | | MET | INS | Sulfonylureas | GLP-1RAs | DPP-4is | SGLT2is | AGis | TDZs | Glinides |
| --- | --- | --- | --- | --- | --- | --- | --- | --- | --- | --- | --- |
| 2013 | | 139 | 96 (69.1%) | 81 (58.3%) | 26 (18.7%) | 2 (1.4%) | 14 (10.1%) | 0 (0%) | 55 (39.6%) | 6 (4.3%) | 8 (5.8%) |
| 2014 | | 120 | 85 (70.8%) | 67 (55.8%) | 36 (30%) | 2 (1.7%) | 25 (20.8%) | 0 (0%) | 59 (49.2%) | 10 (8.3%) | 7 (5.8%) |
| 2015 | | 126 | 87 (69%) | 82 (65.1%) | 30 (23.8%) | 1 (0.8%) | 27 (21.4%) | 0 (0%) | 68 (54%) | 14 (11.1%) | 4 (3.2%) |
| 2016 | | 167 | 117 (70.1%) | 107 (64.1%) | 44 (26.3%) | 3 (1.8%) | 30 (18%) | 0 (0%) | 85 (50.9%) | 15 (9%) | 6 (3.6%) |
| 2017 | | 177 | 146 (82.5%) | 115 (65%) | 35 (19.8%) | 9 (5.1%) | 54 (30.5%) | 1 (0.6%) | 88 (49.7%) | 13 (7.3%) | 6 (3.4%) |
| 2018 | | 180 | 137 (76.1%) | 112 (62.2%) | 34 (18.9%) | 19 (10.6%) | 77 (42.8%) | 7 (3.9%) | 90 (50%) | 9 (5%) | 3 (1.7%) |
| 2019 | | 196 | 150 (76.5%) | 96 (49%) | 36 (18.4%) | 24 (12.2%) | 91 (46.4%) | 47 (24%) | 80 (40.8%) | 9 (4.6%) | 6 (3.1%) |
| 2020 | | 135 | 93 (68.9%) | 70 (51.9%) | 15 (11.1%) | 19 (14.1%) | 48 (35.6%) | 67 (49.6%) | 54 (40%) | 7 (5.2%) | 5 (3.7%) |
| 2021 | | 188 | 141 (75%) | 100 (53.2%) | 19 (10.1%) | 25 (13.3%) | 99 (52.7%) | 116 (61.7%) | 63 (33.5%) | 1 (0.5%) | 4 (2.1%) |

Data are reported as n (%). Abbreviations: T2DM, Type 2 Diabetes Mellitus; TCN: total case number; MET, metformin; INS, insulin; GLP-1RAs, glucagon-like peptide 1 agonists; DPP-4is, dipeptidylpeptidase 4 inhibitors; SGLT2is, sodium glucose co-transporter 2 inhibitors; AGis, alfa glucosidase inhibitors; TDZs, thiazolidinediones;

**Supplementary Table 3 Glucose-lowering medications used in patients with EOT2D and LOT2D**

| Drug class | 2013-2022 | | | 2013-2017 | | | 2018-2022 | | |
| --- | --- | --- | --- | --- | --- | --- | --- | --- | --- |
|  | EOT2D (n=609) | LOT2D (n=981) | P value | EOT2D (n=286) | LOT2D (n=460) | P value | EOT2D (n=336) | LOT2D (n=541) | P value |
| Metformin | 474 (77.8%) | 700 (71.4%) | 0.004 | 220 (78.6%) | 311 (69.3%) | 0.006 | 254 (77.2%) | 389 (73.1%) | 0.181 |
| AGis | 231 (37.9%) | 464 (47.3%) | <0.001 | 119 (42.5%) | 236 (52.6%) | 0.008 | 112 (34.0%) | 228 (42.9%) | 0.010 |
| Sulfonylureas | 99 (16.3%) | 187 (19.1%) | 0.157 | 60 (21.4%) | 111 (24.7%) | 0.307 | 39 (11.9%) | 76 (14.3%) | 0.308 |
| TZDs | 44 (7.2%) | 49 (5.0%) | 0.065 | 30 (10.7%) | 28 (6.2%) | 0.030 | 14 (4.3%) | 21 (3.9%) | 0.824 |
| Glinides | 15 (2.5%) | 40 (4.1%) | 0.087 | 7 (2.5%) | 24 (5.3%) | 0.064 | 8 (2.4%) | 16 (3.0%) | 0.618 |
| DPP-4is | 199 (32.7%) | 344 (35.1%) | 0.329 | 66 (23.6%) | 84 (18.7%) | 0.114 | 133(40.4%) | 260(48.9%) | 0.016 |
| SGLT2s | 140 (23.0%) | 180 (18.3%) | 0.025 | 0 (0%) | 1 (0.2%) | 0.429 | 140(42.6%) | 179(33.6%) | 0.009 |
| GLP-1RAs | 71 (11.7%) | 61 (6.2%) | <0.001 | 10 (3.6%) | 7 (1.6%) | 0.080 | 61(18.5%) | 54(10.2%) | <0.001 |
| Insulin | 370 (60.8%) | 548 (55.9%) | 0.055 | 181 (64.6%) | 271 (60.4%) | 0.246 | 189(57.4%) | 277(52.1%) | 0.124 |

Data are reported as n (%). Abbreviations: EOT2D, early-onset type 2 diabetes; LOT2D, late-onset type 2 diabetes; AGis, alfa glucosidase inhibitors; TZDs, thiazolidinediones; DPP-4is, dipeptidylpeptidase 4 inhibitors; SGLT2is, sodium glucose co-transporter 2 inhibitors; GLP-1RAs, glucagon-like peptide 1 agonists; P values were derived by using the chi-square test.

**Supplementary Table 4 Comparison of medication use between EOT2D and LOT2D, 2013-2021**

| Drug class | Group | 2013 | 2014 | 2015 | 2016 | 2017 | 2018 | 2019 | 2020 | 2021 |
| --- | --- | --- | --- | --- | --- | --- | --- | --- | --- | --- |
| MET | EOT2D | 76.8% | 78.6% | 76.1% | 79.1% | 81.2% | 82.3% | 80.8% | 69.0% | 73.4% |
|  | LOT2D | 63.9% | 66.7% | 65.0% | 64.0% | 83.3% | 72.9% | 73.7% | 68.8% | 76.1% |
| AGis | EOT2D | 28.6% | 50.0% | 52.2% | 38.8% | 46.4% | 43.5% | 29.5% | 35.7% | 31.6% |
|  | LOT2D | 47.0% | 48.7% | 55.0% | 59.0% | 51.9% | 53.4% | 48.3% | 41.9% | 34.9% |
| Sulfonylureas | EOT2D | 16.1% | 26.2% | 17.4% | 25.4% | 21.7% | 12.9% | 17.9% | 11.9% | 10.1% |
|  | LOT2D | 20.5% | 32.1% | 27.5% | 27.0% | 18.5% | 22.0% | 18.6% | 10.8% | 10.1% |
| TDZs | EOT2D | 10.7% | 9.5% | 15.2% | 11.9% | 7.2% | 6.5% | 5.1% | 7.1% | 0 |
|  | LOT2D | 0 | 7.7% | 8.8% | 7.0% | 7.4% | 4.2% | 4.2% | 4.3% | 0.9% |
| Glitinides | EOT2D | 3.6% | 4.8% | 4.3% | 0 | 1.4% | 1.6% | 2.6% | 2.4% | 2.5% |
|  | LOT2D | 7.2% | 6.4% | 2.5% | 6.0% | 4.6% | 1.7% | 3.4% | 4.3% | 1.8% |
| DPP-4is | EOT2D | 14.3% | 26.2% | 23.9% | 20.9% | 31.9% | 41.9% | 38.5% | 23.8% | 48.1% |
|  | LOT2D | 7.2% | 17.9% | 20.0% | 16.0% | 29.6% | 43.2% | 51.7% | 40.9% | 56.0% |
| SGLT2is | EOT2D | 0 | 0 | 0 | 0 | 0 | 4.8% | 32.1% | 54.8% | 68.4% |
|  | LOT2D | 0 | 0 | 0 | 0 | 0.9% | 3.4% | 18.6% | 47.3% | 56.9% |
| GLP-1RAs | EOT2D | 3.6% | 2.4% | 0 | 1.5% | 8.7% | 8.1% | 20.5% | 16.7% | 22.8% |
|  | LOT2D | 0 | 1.3% | 1.3% | 2.0% | 2.8% | 11.9% | 6.8% | 12.9% | 6.4% |
| INS | EOT2D | 64.3% | 54.8% | 73.9% | 62.7% | 66.7% | 67.7% | 48.7% | 54.8% | 55.7% |
|  | LOT2D | 54.2% | 56.4% | 60.0% | 65.0% | 63.9% | 59.3% | 49.2% | 50.5% | 51.4% |

Data are reported as n (%). Abbreviations: EOT2D, early-onset type 2 diabetes; LOT2D, late-onset type 2 diabetes; MET, metformin; INS, insulin; GLP-1RAs, glucagon-like peptide 1 agonists; DPP-4is, dipeptidylpeptidase 4 inhibitors; SGLT2is, sodium glucose co-transporter 2 inhibitors; AGis, alfa glucosidase inhibitors; TDZs, thiazolidinediones;

**Supplementary Table 5 Monotherapy or combined therapy in EOT2D and LOT2D groups**

|  | EOT2D (n=609) | LOT2D (n=981) | P value |
| --- | --- | --- | --- |
| Monotherapy | 89 (14.6%) | 139 (14.2%) | 0.806 |
| Using two drugs | 188 (30.9%) | 309 (31.5%) | 0.793 |
| Using three or more drugs | 331 (54.4%) | 520 (53.0%) | 0.601 |

Data are reported as n (%). Abbreviations: EOT2D, early-onset type 2 diabetes; LOT2D, late-onset type 2 diabetes; P values were derived by using the chi-square test.

**Supplementary Table 6. Comparison of combined therapy used in 2013 and 2021**

|  | Prescription number (Relative percentage) | |
| --- | --- | --- |
| Year | 2013 | 2021 |
| Patients using Combined therapy | 104 | 172 |
| MET+insulin | 26 (25.0%) | 4 (2.3%) |
| AGis+insulin | 14 (13.5%) | 1 (0.6%) |
| MET+Sulfonylureas | 12 (11.5%) | 0 |
| MET+AGIs | 7 (6.7%) | 2 (1.2%) |
| MET+DPP-4is  MET+SGLT2is | 4 (3.9%)  0 | 11 (6.4%)  9 (5.2%) |
| MET+DPP-4is+SGLT2is | 0 | 16 (9.3%) |
| MET+DPP-4is+SGLT2is+insulin | 0 | 15 (8.7%) |
| Contained |  |  |
| MET | 83 (79.8%) | 139 (80.8%) |
| AGis | 48 (46.1%) | 62 (36.0%) |
| Sulfonylureas | 26 (25.0%) | 19 (11.0%) |
| TZDs | 6 (5.7%) | 1 (0.5%) |
| Glinides | 8 (7.6%) | 4 (2.3%) |
| DPP-4is | 14 (13.4%) | 99 (57.5%) |
| SGLT2is | 0 | 114 (66.2%) |
| GLP-1RAs | 2 (1.9%) | 23 (13.3%) |
| Insulin | 66 (63.4%) | 92 (53.4%) |

Data are reported as n (%). Abbreviations: MET, metformin; AGis, alfa glucosidase inhibitors; TDZs, thiazolidinediones; DPP-4is, dipeptidylpeptidase 4 inhibitors; SGLT2is, sodium glucose co-transporter 2 inhibitors; GLP-1RAs, glucagon-like peptide 1 agonists
